# Effects of Invasive European Fire Ants (*Myrmica rubra*) on Herring Gull (*Larus argentatus*) Reproduction

**DOI:** 10.1371/journal.pone.0064185

**Published:** 2013-05-17

**Authors:** Luke E. DeFisher, David N. Bonter

**Affiliations:** 1 Cornell University, Department of Ecology and Evolutionary Biology, Ithaca, New York, United States of America; 2 Cornell Lab of Ornithology, Ithaca, New York, United States of America; Stanford University, United States of America

## Abstract

Various invasive ant species have negatively affected reproductive success in birds by disrupting nest site selection, incubation patterns, food supply, and by direct predation on nestlings. Impacts can be particularly severe when non-native ants colonize seabird nesting islands where thousands of birds may nest in high densities on the ground or in burrows or crevices. Here we report on the first documented effects of *Myrmica rubra*, the European fire ant, on the reproduction of birds in its non-native range. We documented herring gulls (*Larus argentatus*) on Appledore Island, Maine, engaging in more erratic incubation behaviors at nests infested by the ants. Newly-hatched chicks in some nests were swarmed by ants, leading to rapid chick death. Due to high overall rates of chick mortality, survival probabilities did not vary between nests with and without ant activity, however chick growth rates were slower at nests with ants than at ant-free nests. Ant infestation likely leads to longer-term fitness consequences because slower growth rates early in life may ultimately lead to lower post-fledging survival probabilities.

## Introduction

The effects of invasive organisms on native species are of growing concern as globalization leads to the translocation of species and biotic homogenization [1], [2]. Invasive ants are particularly disruptive to native communities, with five species of ants listed among 100 of the worst invasive species [3]. Globally, several ant species have impacted the reproductive success of birds that follow a diversity of nesting strategies. Although native ants can potentially affect the reproductive success of birds [4], recent research demonstrates widespread effects of non-native ants. Island-nesting birds may be particularly vulnerable to population decline due to the limited availability of suitable nesting islands and the potential development of super-colonies of non-native ants following invasion [5], [6]. In some species, the presence of the ants can deter a breeding pair from starting a nest [7], [8]. In other cases, birds are able to establish a nest but are disturbed by ant activity during the incubation period. This can result in metabolic costs to the incubating adults and reduced incubation efficiency [9]. Numerous studies document the effects of invasive ants on newly-hatched chicks, when birds are most vulnerable and swarming by ants can lead to chick death or nest abandonment by adults [5], [10]–[12]. Even in the absence of direct chick morality attributed to ant activity, ants at the nest can negatively affect chick growth rates, potentially compromising the long-term survival of the chick [13].

Extensive research has documented the negative effects of red imported fire ants (*Solenopsis invicta*) on land birds in North America [12], [14]–[19]. Other non-native ant species have caused nest abandonment, lower hatching success, and chick death in seabird colonies on islands in the Indian and Pacific Oceans. In the Seychelles, the crazy ant (*Anoplolepis longipes*) excluded sooty terns (*Sterna fuscata*) from parts of the nesting colony, and caused chick mortality in white terns (*Gygis alba*) [8]. In the Hawaiian islands, tropical fire ants (*Solenopsis geminata*) attacked wedge-tailed shearwater (*Puffinis pacificus*) chicks, causing them to either lose weight or gain weight at a slower rate than uninjured chicks [11].

Since the first record of its appearance in Boston, Massachusetts, USA in 1902, the European fire ant, *Myrmica rubra*, has been detected in various coastal areas in northeastern North America from Quebec to New Jersey [20]. Research conducted on Mt. Desert Island, Maine, found that *M. rubra* had a measurable impact on native invertebrate communities and was capable of killing small mammals [21]. Other vertebrates unable to escape rapidly, like birds reliant upon a fixed nesting location, may also be vulnerable to disturbance by the ants. Although it has not been documented previously, *M. rubra* has the potential to negatively impact native bird communities by influencing reproductive success.

Our objective was to quantify the potential effects of *M. rubra* on the reproductive behavior and success of birds in the ants' non-native range. Our study was motivated by previous anecdotal observations of *M. rubra* swarming day-old herring gull (*Larus argentatus*) hatchlings, and by the general lack of information on the effects of *M. rubra* on avian populations. We focused on Appledore Island, Maine, where gulls nest in high densities [22] and *M. rubra* is patchily distributed. We predicted that (1) ant presence in the nest would alter the natural incubation behaviors of gulls, and (2) chicks at nests affected by ant activity would suffer greater mortality rates and slower growth rates than chicks at ant-free nests.

## Methods

### Ethics statement

This research was carried out in strict accordance with the Guidelines for the Use of Wild Birds in Research of the Ornithological Council and approved by the Cornell University Institutional Animal Care and Use Committee (protocol #2011-0036). Research was conducted on property managed by Cornell University for research and educational purposes. This work did not involve any threatened, endangered or protected species. No vertebrates were collected or sacrificed for this study.

### Study site

**Figure 1:**
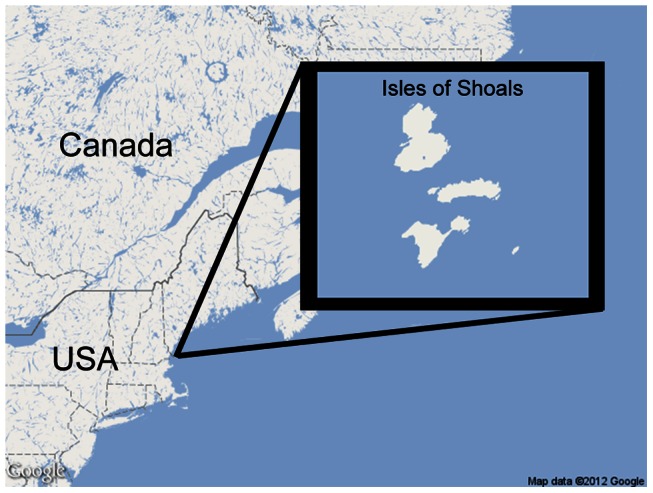
*Myrmica rubra* colonized Appledore Island is the Isles of Shoals (northernmost island shown), 10 km off the coast of New Hampshire, USA. The island hosts hundreds of nesting herring gulls.

The study was conducted at Shoals Marine Laboratory, Appledore Island, in the Gulf of Maine (Fig. 1, 42°59′N 70°37′W). Located approximately 10 km off the coast of Portsmouth, New Hampshire, USA, Appledore Island is the largest of a nine-island archipelago. It has supported breeding colonies of herring gulls and great black-backed gulls (*Larus marinus*) since at least the first quarter of the twentieth century [23]. Although the native range of *M. rubra* extends throughout northern Europe from Great Britain to western Siberia, the ant has been observed on Appledore Island since at least the 1970s (J. Kingsbury, personal communication).

### Preliminary measurement of ant distribution

We recorded *M. rubra* distribution using bait traps with approximately 4 cm^3^ of peanut butter spread onto a 20 cm^2^ piece of paper. We established a 24 m square grid of the island in accessible areas suitable for nesting gulls and placed baits on the ground for 20 minutes following standard ant sampling methods [24]. In cases where baits were set in close proximity to a gull nest, a chicken wire cage was placed over the trap to prevent gulls from eating the traps but allowing ants to access the bait. Gulls nesting in areas of the islands with active *M. rubra* colonies were identified as potential focal birds for the remainder of the study.

### Gull behavior and reproduction

Incubation behavior was monitored at 35 nests located in a microhabitat suitable for *M. rubra*. Appropriate microhabitat included sites with sufficient soil accumulation, in downed woody debris, at the base of trees, and among the roots of vegetation [20], [25]. Observations of incubating adults (30 minutes, N = 42 total observation periods) were initiated after the focal nest was checked for the presence of *M. rubra*. Behavioral observations were recorded every minute with behavior classified into six categories: undisturbed incubation, preening, resettling, shaking, off nest, and other. Observations were initiated at various times throughout the day between May 22 and June 10, 2011.

To monitor chick growth and survival, we labeled 39 herring gull nests in areas with known *M. rubra* activity during the early incubation period in May 2011. We recorded nest contents and the presence/absence of ants during daily nest checks from May 22–July 16 (when the chicks approached fledging age). Eggs typically hatch asynchronously, and chicks were marked as A, B, or C chick, depending on hatch order. To quantify growth rates, we measured chick weight and the length of the head plus the upper mandible (head+bill) for each chick on days 1, 3, 5, 7, and 9 after hatching.

### Statistical Analyses

To test for the potential effect of ants on incubation behavior, we classified observations of incubating birds into a binary response including either undisturbed incubation behavior (0) or any other behavior that indicated a potential response to ant activity (1). Such responses included preening, stepping off of the nest, resettling on the nest, or head shaking. Because multiple observations were recorded for each individual incubating gull, the response was coded as the proportion of minutes that the individual engaged in behaviors other than undisturbed incubation. Explanatory variables included the presence or absence of ant activity at the nest immediately prior to the observations (binary), and local temperature and humidity at the start of the observation period. We used logistic regression in SAS (PROC LOGISTIC, [26]) for the analysis.

We quantified differences in chick growth between nests with ants and ant-free nests using a mixed model where growth was modeled as a function of the presence of ants while controlling for chick age (continuous variable). Hatch order within the nest (categorical, A, B, or C chick) was also included in the model because the first chick to hatch (A) typically grows at a faster rate than later-to-hatch siblings. Individual chicks were measured repeatedly, so chick identification was used as a random variable in the model. Further, siblings represent repeated measures from the same nest, so nest identification was also used as a random variable. Ant activity at each nests was assessed once daily during regular nest checks. If ants were recorded at the nest at any point during the first 5 days post-hatching, the nest was categorized as an “ant” location. After the first 5 days post-hatching, the chicks became mobile and could move around the parents' territory and evade ants if necessary.

To test for differences in chick survival between nests with and without ants, we compared the proportion of chicks surviving to day 5 using logistic regression (PROC LOGISTIC) with chick survival (1) or mortality (0) related to the presence or absence of ants. Because the detection probability of chicks through day 5 is perfect, mark-recapture survival analyses were not necessary.

## Results

Incubation observations were conducted for 1260 minutes at 35 herring gull nests (20 with ant activity). Birds incubating at nests with ant activity engaged in more preening and other behaviors disrupting incubation than birds at nests without ant activity (Table 1, χ^2^_1_ = 17.49, *p*<0.001). We detected a weak inverse relationship between temperature and bird activity (χ^2^_1_ = 5.77, *p* = 0.016, r = 0.13), but humidity did not influence incubation behavior (χ^2^_1_ = 0.92, *p* = 0.336).

**Table 1:** Proportion of time and relative investment of time spent for birds at nests with ant activity and nests without ant activity.

	Proportion of Time		Relative Investment^1^
Activity	Ants	No Ants	Ants/No Ants
Undisturbed Incubation	0.843	0.927	0.909
Preening	0.058	0.035	1.674
Shaking	0.015	0.002	9.900
Off Nest	0.005	0.002	3.300
Resettling	0.030	0.032	0.943
Other	0.048	0.003	15.950

1 Relative investment is calculated as the proportion of time spent by birds in nests with ants divided by the proportion of time spent by birds in nests without ants.

**Figure 2:**
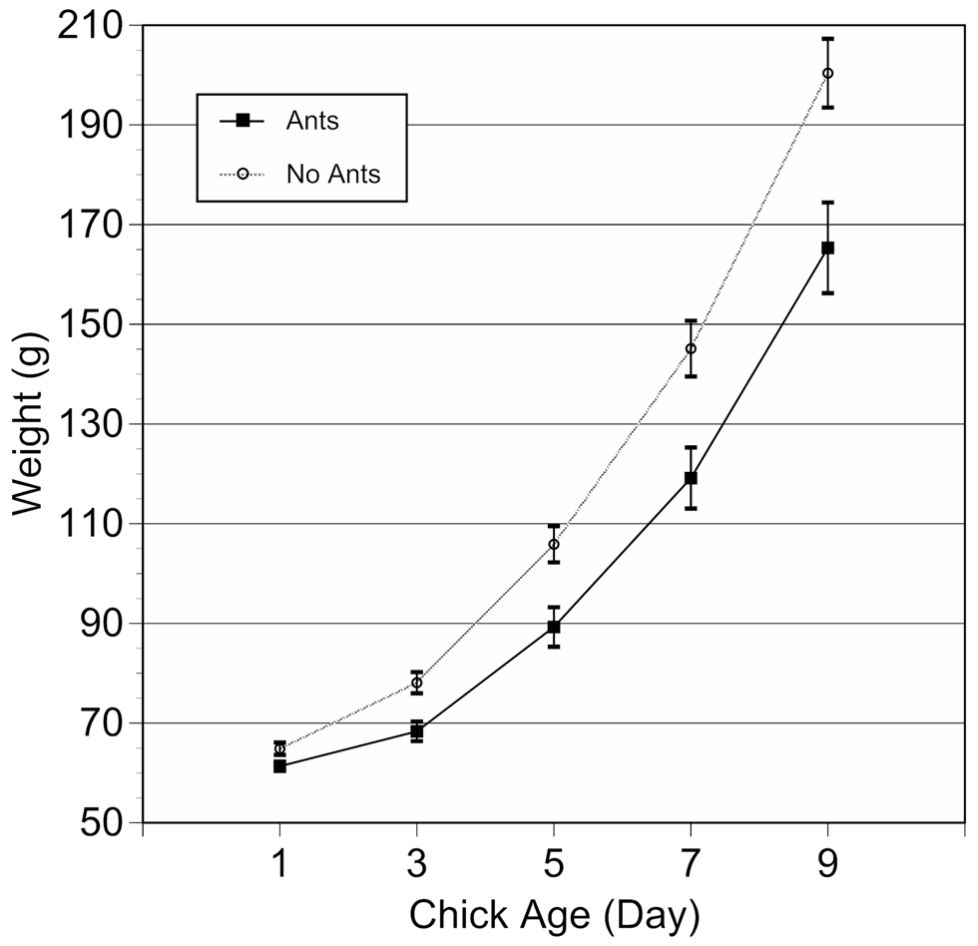
Herring gull chicks in nests without *M. rubra* activity grew at a faster rate than chicks in nests with the ants (mean ± standard error).

Chick growth was measured for 81 chicks located in 39 nests from the vegetated areas of the island (17 with ant activity). Chick weight was influenced by the presence of ants in the nest (F_1,271_ = 8.50, *p* = 0.004) after controlling for chick age (F_1,271_ = 1144.9, *p*<0.001) and position in the hatching order (F_2,271_ = 8.94, *p*<0.001). On average, chicks in nests without ant activity grew more rapidly than did chicks in nests with ant activity (Fig. 2), chicks gained weight with age, and older chicks weighed more than younger siblings. The same differences in growth were detected using a measure of structural size (head+bill length), with the presence of ants (F_1,271_ = 4.10, *p* = 0.044), chick age (F_1,271_ = 2521.6, *p*<0.001), and position in the hatching order (F_2,271_ = 5.16, *p* = 0.006) all effecting size.

No differences were detected in chick survival rates through day 5 as a function of the presence or absence of ants at the nest (χ^2^_82_ = 0.04, *p* = 0.85). The proportion of chicks that died in the first 5 days was nearly identical at nests with ants (0.16) as at ant-free nests (0.17). Ants were observed swarming over newly hatched chicks in five nests. Swarming was concentrated around the bill and the eyes of the chicks, with the skin around the eyes often swollen and red in color. Chicks responded to the ants by shaking their heads and stomping their feet. When handled, some chicks were listless and unable to support their head. Of the twelve chicks affected in the swarmed nests, six were found dead in the nest within two days of the initial observations and six survived.

## Discussion

Our study is the first to quantify the negative influence of *M. rubra* on bird reproduction within the introduced range of the ant. Newly hatched herring gull chicks in nests with ant activity grew at a significantly slower rate than chicks in nests without ant activity, a pattern detected in previous studies examining the effects of invasive ant species on chick development. Body mass of northern bobwhite (*Colinus virginianus*) chicks was lower for birds experimentally exposed to *S. invicta* than for unexposed chicks [10]. In the Hawaiian archipelago, Plentovitch et al. [11] examined the impact of the invasive big headed ant (*Pheidole megacephala*) and *S. geminata* on wedge-tailed shearwater colonies. Chicks with severe injuries caused by *S. geminata* weighed less than chicks sustaining moderate, mild, or no ant-induced injuries, and severely injured chicks either lost weight or grew at a slower rate than uninjured chicks. In another study in Finland, the red wood ant (*Formica rufa*), although native to the area, depressed the food supply and interfered with the foraging behavior of Eurasian treecreepers. Because of the indirect effect of the ant on territory quality, body mass for nestling treecreepers was lower for breeding pairs whose ranges overlapped with the ant [27].

Despite differential growth rates and six incidents of chick mortality attributed to ant activity in our study, chick survival rates in the first five days after hatching were nearly identical at infested nests as at ant-free nests. Mortality rates are generally high in young larids due to predation, limited food, harsh weather and other factors [28]. The additional mortality introduced by the ants did not influence the overall survival rates of chicks through 5 days of age. Similarly, Plentovitch et al. [11] concluded that the big headed ant had no impact on the hatching or fledging success of the wedge-tailed shearwater colony, contrary to their own unpublished observations. *M. rubra* may nonetheless impact long term survival of affected chicks through a reduction in growth rates. Evidence from previous studies of gull biology suggests that faster-growing chicks survive better after fledging [13], [28]. Faster-growing chicks will be larger and can outcompete conspecifics for food resources during the first winter following independence. Because it is the period of highest post-fledgling mortality, enduring the first winter is critical to the long-term survival of young gulls. Therefore, by reducing chick growth rates, exposure to *M. rubra* during the initial days of life may put juvenile herring gulls from infested nests at a lasting competitive disadvantage.

Incubating herring gulls engaged in significantly more erratic behavior when ant activity was observed at the nest. Adult birds preened frequently, shook their wings and bills, and frequently left and resettled on the nest. This and other erratic behavior, such as scratching, pecking, and hopping, has been documented in other incubating birds exposed to aggressive invasive ants [8], [9], [11], [29], [30]. Erratic incubation behavior caused by the ants may hinder embryonic development, which requires consistent incubation to warm the egg above a critical temperature [31]. Invasive ants are also known to negatively influence reproduction by increasing the energy expenditure of the parent [11]. For instance, *S. invicta* was found to increase the metabolic cost of nest defense in a tree-nesting songbird [9] with potential longer-term effects on reproductive success. Although we did not quantify energy expenditure in the current study, adult herring gulls provoked into erratic behavior by *M. rubra* may expend more energy during incubation than gulls in ant-free habitats, ultimately leading to reduced parental investment in chick rearing.

Although *M. rubra* had an impact on adult behavior while incubating, it did not affect nest attendance. Nest attendance is critical during the incubation period because it reduces the exposure of the eggs to potential predation [31]. In other ecosystems, invasive ants have caused adult birds to abandon their nests [8], [12], [15]. No nest site abandonment was observed in any of the nests in our sample, however, and other species of birds have been able to incubate in the presence of an invasive ant without any apparent disturbance [18].

Considering that the majority of chick rearing on Appledore Island takes place in areas absent of *M. rubra*, the impact of the ant on the reproductive success of the gull colony as a whole is likely quite low. The majority of herring gulls build their nest on the rocky exterior of the island at locations unsuitable for *M. rubra* nesting and foraging. Other birds with broader nesting habitat niche overlap with *M. rubra* could be more vulnerable to the effects of the ants if this invasive species continues to spread and colonizes additional nesting islands. Leach's Storm-Petrels (*Oceanodroma leucorhoa*), for instance, nest in underground burrows in habitats that would be attractive to *M. rubra* if the ant were introduced to islands in the region with storm-petrel nesting colonies.

In sum, we found that the invasive *M. rubra* can cause erratic behavior by incubating adult gulls in the ants' non-native range. Chick growth rates were lower in nests with ant activity, but we observed no impact on short-term survival of chicks between nests with and without ant activity. Although the bulk of the herring gull colony is not significantly affected by the ants, this is the first study to quantitatively demonstrate that *M. rubra* can have a negative effect on individual birds in the introduced range. As the range of this invasive ant continues to expand, future studies should examine its impact on other avian populations.
